# Pyomyositis associated with chemotherapy for endometrial cancer: a case report

**DOI:** 10.1186/1477-7819-11-45

**Published:** 2013-02-25

**Authors:** Yoshifumi Nakao, Masatoshi Yokoyama, Satoshi Nishiyama, Mariko Hashiguchi, Satomi Aihara, Makio Yasunaga, Mitsuyo Noguchi, Tsuyoshi Iwasaka

**Affiliations:** 1 Department of Obstetrics and Gynecology, Faculty of Medicine, Saga University, Nabeshima 5-1-1, Saga, Saga 8498501, Japan; 2 Department of Obstetrics and Gynecology, Nagasaki Prefecture Izuhara Hospital, Izuhara-cho Higashizato 303-1, Tsushima, Nagasaki, 8170016, Japan; 3 Department of Obstetrics and Gynecology, Saga Prefectural Hospital KOSEIKAN, Mizugae 1-12-9, Saga, Saga, 8408571, Japan; 4 Department of Obstetrics and Gynecology, Kohoukai Takagi Hospital, Sakemi 141-11, Ookawa, Fukuoka, 8310016, Japan

**Keywords:** Pyomyositis, Endometrial carcinoma, Chemotherapy

## Abstract

Pyomyositis is a rare complication of chemotherapy for non-hematological malignancies. A 58-year-old woman with endometrial carcinoma, in whom pyomyositis developed during adjuvant chemotherapy, was presented in this report. After initiating empiric antibiotic therapy for febrile neutrocytopenia, screening CT showed multiple abscesses in the lower limbs. Operative drainage of the abscess was effective.

## Background

Pyomyositis is originally known as an infectious disease of the large skeletal muscles, which is common in tropical areas and often results in abscess formation and sepsis. In patients with pyomyositis, *staphylococcus aureus* is detected more often than *streptococcus* and other bacteria. Important factors of pyomyositis are reported to be local mechanical trauma, parasitic infections, and malnutrition. Furthermore, it is recently reported that pyomyositis is associated with the immunocompromised host due to infection with human immunodeficiency virus (HIV), hematological malignancies, and neutropenia secondary to oncologic chemotherapy [1].

To date, pyomyositis developing as a complication of chemotherapy in patients with gynecologic malignancy has been rarely reported [2]. In this report, we presented a patient with pyomyositis who had received systemic chemotherapy for endometrial carcinoma.

## Case presentation

A 58-year-old previously healthy Japanese woman (body mass index 22.1) presented sudden massive postmenopausal bleeding, and diagnosed endometrial carcinoma after gynecological, pathological, and radiological examinations. She did not have family history of cancer. She underwent total abdominal hysterectomy, bilateral salpingo-oophorectomy, pelvic lymphadenectomy, and sampling of para-aortic lymph nodes. Histopathology revealed endometrioid adenocarcinoma grade 1. Tumor cells involved the serosa of uterine corpus. The pathological diagnosis of endometrial carcinoma stage IIIc, T3a N1 (right obturator lymph node) M0, was made. The patient was planned to be given whole pelvic external irradiation followed by systemic chemotherapy.

**Figure 1:**
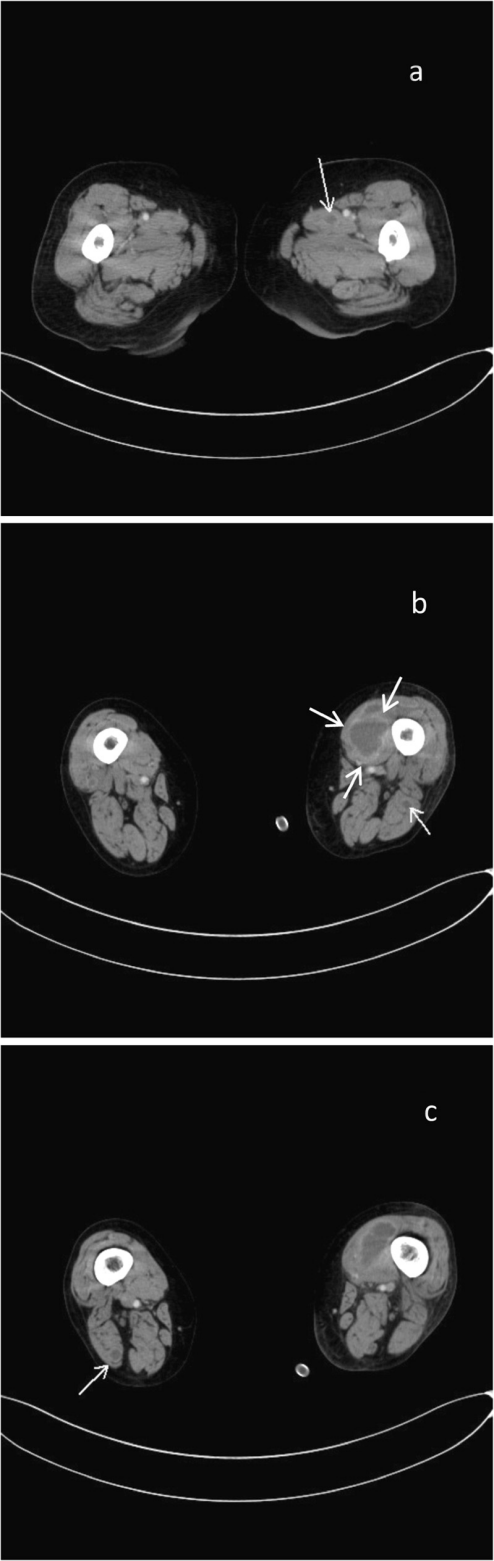
Multiple abscesses in the lower extremities showing low density areas with enhancement (arrows).

External radiotherapy was delivered with an 18-MV photon beam for 5 days each week. Whole pelvic irradiation was delivered to a total dose of 45 Gy in 25 fractions. The first course of chemotherapy consisted of docetaxel (70 mg/m2) and carboplatin (AUC = 5) administrated on Day 0. On Day 11, she presented at our outpatient clinic with worsened general fatigue, appetite loss, and restlessness. Her body temperature developed to 39.1°C. Physical examination showed muscular weakness and proximal dominant lower limb muscle ache. Routine blood test revealed decreased white blood cell count (1.6 × 10^3^ /mcL), decreased platelet count (5.3 × 10^4^ /mcL), increased C-reactive protein (46.14 mg/dL (normal, 0 to 0.3)), increased serum CPK (8152 IU/L), increased LDH (638 IU/L), increased serum creatinine (2.12 mg/dl) and increased blood urea nitrogen (44.0 mg/dl). Imaging of her head and chest-abdomen with computed tomography showed no remarkable changes. Empirical anti-bacterial chemotherapy for pyrogenic leukocytopenia with cefepime (4 g/day) was started. She was found to have *Streptococcus dysgalactiae equisimilis* bacteremia. She received intravenous antimicrobial treatment with vancomycin (2 g/day) and clindamycin (1.8 g/day); which improved her conditions. However, her body temperature developed to 39.7°C on Day 14 again, and prolonged fever elevation maintained at 38 to 39°C. A repeated physical examination showed tenderness on left medial thigh. CT scan demonstrated the formation of multiple abscesses in the both femoral muscles on Day 21 (Figure 1).

The patient underwent open surgery. The incision was made in the left medial thigh and opened the fascia. Further incision was made in the swelling medial vastus muscle. About 50 mL of pus was drained. A Penrose drain was left in the abscess and the incision was closed. The drained pus showed the same bacteria with the bacteremia. The fever decreased and general conditions improved. In addition, 7 weeks oral administration of clindamycin (1.2 g/day) and levofloxacin (0.5 g/day) suppressed the relapse of inflammation.

Because of these severe complications, an additional course of chemotherapy was not given. After 40 months of this event, this patient is still alive with the disease.

## Discussion

Bacterial pyomyositis is a purulent infection of the large skeletal muscle, and is most commonly involved in the gluteal areas and the thigh; which results in abscess formation. It is an endemic disease in the tropics, and seems to occur only occasionally in the temperate zones. However, pyomyositis is increasingly recognized in non-tropical areas as one of the complications in the immunocompromised host associated with HIV infection, diabetes mellitus, and chemotherapy-induced neutropenia. Both malignant and non-malignant hematological diseases have been reported in association with pyomyositis [1,3]. A review paper reports 45 patients with hematological neoplastic diseases associated with pyomyositis [1]. Pyomyositis is an uncommon complication of chemotherapy for non-hematological malignancies. To our knowledge based on MEDLINE, only nine cases of solid tumor-related pyomyositis have been reported (Table 1). Among them, two have retroperitoneal malignant teratomas [4], two lung cancer [5,6], two breast cancer [3,7], one colon cancer [3], one glottic cancer [3], and one endometrial cancer [2].

**Table 1:** Review of patients with pyomyositis associated with solid malignancies

	**Patient (age/sex)**	**The origin of cancer**	**Chemotherapy**	**Site of pyomyositis**	**Surgical drainage**	**Bacteria**	**Reference**
1	43/M	Retroperitoneal teratoma	VCR, BLM, CDDP, MTX, VP-16, CPA	Psoas muscle	Done	Bacteroides fragilis	[4]
2	40/M	Retroperitoneal teratoma	VCR, BLM, CDDP, MTX, VP-16, CPA	Psoas muscle	Done	Bacteroides fragilis	[4]
3	67/M	Lung	CDDP, VDS, MMC	Lower limb	Done	Staphylococcus aureus	[5]
4	47/F	Breast	5-FU, DXR, CPA	Carf	Done	Staphylococcus aureus	[7]
5	62/M	Lung	AMR, VNR	Upper arm, lower limb	Done	Staphylococcus aureus	[6]
6	NR	Breast	Unknown	NR	NR	NR	[3]
7	NR	Colon	Unknown	NR	NR	NR	[3]
8	NR	Glottis	Unknown	NR	NR	NR	[3]
9	58/F	Endometrium	CBDCA, PTX	Gluteus medius	CT-guided drainage	Staphylococcus aureus	[2]
10	58/F	Endometrium	CBDCA, DTX	Lower limb	Done	Streptococcus dysgalactiae equisimilis	This case

*AMR *Amrubicin; *BLM *Bleomycin; *CBDCA *Arboplatin; *CDDP* Cisplatin; *CPA *Cyclophosphamide; *DTX *Docetaxel; *DXR* Doxorubicin; *MMC *MitomycinC; *MTX *Methotrexate; *NR *Not recorded; *PTX *Paclitaxel; *VNR *Vinorelbine; *VP-16 *Etoposide; *VCR *Vincristine; *VDS *Vindesine.

Primary pyomyositis is believed to be caused by transient bacteremia, because it develops without an obvious penetrating injury or any other clear portals of entry in the majority of cases [3]. Development of pyomyositis in patients with neoplastic diseases after chemotherapy is usually ascribed to neutropenia and/or immunodeficiency caused by cancer. However, subclinical myopathy secondary to malignancy and/or its treatment is another possible cause [3]. Several chemotherapeutic drugs including anthracyclines, vinca alkaloids, and corticosteroids have been reported to induce muscle toxicity-related pyomyositis [7,8]. It is recently reported that a woman with endometrial cancer develops pyomyositis after the first cycle of carboplatin and paclitaxel [2]. Our patient also received docetaxel and dexamethasone. However, the muscle toxicity of docetaxel has never been reported. Whether pyomyositis observed in the above two cases was related to the drug was unclear.

Diagnosis of pyomyositis is facilitated by ultrasonography, MRI, or CT scanning of the affected area, and by aspiration of fluid for microbiological testing. According to previous literature, *Staphylococcus aureus* is the most common cause of pyomyositis. Several other bacteria including Gram-negative organisms, other Gram-positive organisms than S*taphylococcus aureus* (predominantly *Streptococcus*), anaerobes, mycobacteria, and fungi are implicated as a cause of pyomyositis [3].

Early diagnosis enables complete drainage of purulent materials and successful treatments, and leads to resolution in the vast majority of cases. The immune status of the host, clinical courses, and the number of abscess should be considered when determining the length of the treatment for pyomyositis.

## Conclusion

We described an unusual case of pyomyositis associated with chemotherapy for gynecologic malignancy. This should be kept in mind in the differential diagnosis of febrile neutropenia.

## Consent

Informed consent was obtained from the patient for publication of this case report and accompanying images. A copy of the written consent is available for review by the Editor-in-Chief of this journal.

## Competing interests

The authors declare that they have no competing interests.

## Author’s contributions

YN contributed mainly in designing, literature review, and writing work. MY, MH, and MN have operated this case. SN, SA, MY, and TI gave advices and edited the discussion. All authors read and approved the final manuscript.
